# Diffuse Alveolar Damage of the Lungs in Forensic Autopsies: Assessment of Histopathological Stages and Causes of Death

**DOI:** 10.1100/2012/657316

**Published:** 2012-09-17

**Authors:** Halide Nur Urer, Gokhan Ersoy, Emine Dilek Yılmazbayhan

**Affiliations:** ^1^Department of Pathology, Yedikule Teaching Hospital for Chest Diseases and Thoracic Surgery, 34020 Istanbul, Turkey; ^2^Department of Forensic Pathology, Istanbul University, Institute of Forensic Medicine, 34452 Istanbul, Turkey; ^3^Department of Pathology, Istanbul Medical Faculty, Istanbul University, 34452 Istanbul, Turkey

## Abstract

*Introduction*. Diffuse alveolar damage (DAD) is a morphological prototype of acute interstitial pneumonia. Hospital autopsies or open-lung biopsies are used to monitor common alveolar damage and hyaline membrane (HM) development histopathologically. The aim of this study was to detect histopathological profiles and frequency of DAD and HM in adult forensic autopsies. *Materials and Methods*. In total, 6813 reports with histopathological samples in 12,504 cases on which an autopsy was performed between 2006 and 2008 were investigated. Sixty-six individuals >18 years of age who were diagnosed with DAD were included. Hematoxylin- and eosin-stained lung preparations were reexamined in line with the 2002 American Thoracic Society/European Respiratory Society idiopathic interstitial pneumonia consensus criteria. *Results*. Histopathological examination revealed that 50 cases (75.7%) were in the exudative phase and 16 (24.2%) were in the proliferative phase. Only the rate of alveolar exudate/oedema in exudative phase cases (*P* = 0.003); those of alveolar histiocytic desquamation (*P* = 0.037), alveolar fibrosis (*P* = 0.017), chronic inflammation (*P* = 0.02), and alveolar fibrin (*P* = 0.001) in proliferative cases were significantly higher. The presence of alveolar fibrin was the only independent variable in favour of proliferative cases (*P* = 0.016). *Conclusion*. The detection of all DAD morphological criteria with the same intensity is not always possible in each case. Forensic autopsies may provide a favourable means for expanding our knowledge about acute lung damage, DAD, and interstitial lung disease.

## 1. Introduction 

Diffuse alveolar damage (DAD) is the morphological prototype of acute interstitial pneumonia and is characterised by a rapid and fatal clinical course. DAD manifests clinically as acute respiratory distress syndrome (ARDS). It can be seen in sepsis, shock, trauma, severe ARDS, and idiopathic cases with undetected aetiological factors as well as acute exacerbations of chronic interstitial lung diseases [1–3]. While diffuse bilateral opacity is observed on lung radiology, many cases display deep hypoxemia that requires mechanical ventilation; the mortality rate is 43–50% [4, 5].

In addition to experimental animal studies, hospital autopsies or open-lung biopsies are needed to histopathologically monitor common alveolar damage and hyaline membrane (HM) development. A reduction in the rate of alveolar damage has been known for a long time [6, 7]. Due to the high mortality and morbidity risk during the acute phase of the disease, late or organising stage DAD is identified generally in the pathological samples of individuals undergoing open lung biopsy [8, 9]. An open biopsy may trigger acute attacks in patients with idiopathic pulmonary fibrosis [10]. Given the restrictions when performing an open-lung biopsy in patients with acute and severe respiratory distress, a limited number of studies have examined biopsy data from this patient group [11–13]. Thus, forensic autopsies and the examination of DAD prevalence may be an alternative area of research [14, 15].

The aim of this study was to detect DAD and HM frequency in adult forensic autopsies, examine the histopathological profiles of these cases, and identify other accompanying histopathological changes to determine the characteristics of cases in which these assessments were made and to recognise the case spectrum that we may encounter in future forensic autopsies.

## 2. Materials and Methods

**Figure 1:**
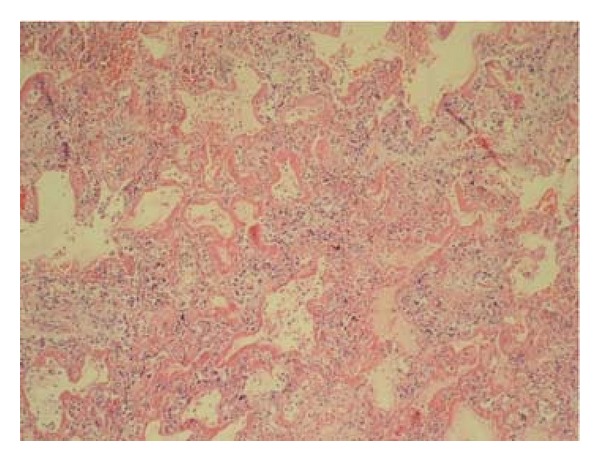
Prevalent hyaline membrane, H&E ×100.

**Figure 2:**
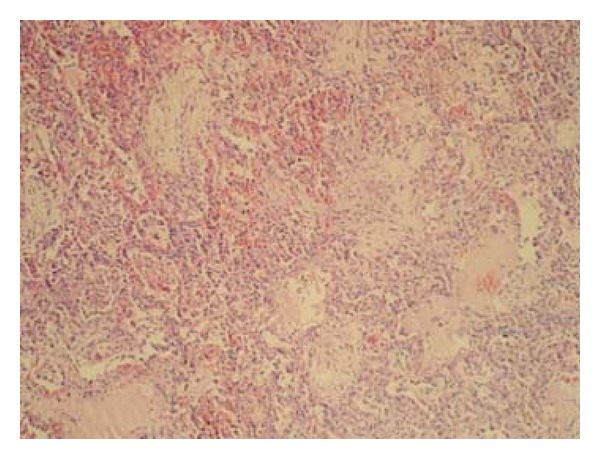
Alveolar organizing fibrosis, H&E, ×100.

We reviewed 6813 reports with histopathological samples in 12,504 cases on which an autopsy was performed between 2006 and 2008 at the Ministry of Justice, Institute of Forensic Medicine, Morgue Specialization Department. Sixty-six individuals >18 years of age and diagnosed with DAD were included. Demographic information and cause of death were recorded from the autopsy reports. Hematoxylin- and eosin- (H&E-) stained lung samples obtained from each case were evaluated again by a pathologist (HNU) under a light microscope. During this evaluation, cases were examined in line with the 2002 American Thoracic Society/European Respiratory Society (ATS/ERS) idiopathic interstitial pneumonia consensus criteria (Table 1) [16]. Among the lesions defined in the criteria, alveolar acute inflammation, neutrophilic abscess, and histiocytes were graded for the intensity of alveolar epithelial cell desquamation, eosinophilia, and alveolar chronic inflammation, whereas other lesions as HM (Figure 1) were graded for diffusion. Intensity and diffusion parameters were ranked as (0) none, (1) mild, (2) medium, or (3) severe intensity/diffusion. The presence of any alveolar organizing fibrosis (Figure 2) was accepted as the main criterion to distinguish exudative from proliferative phases [17]. Causes of death were classified according to DAD phase.

Fisher's exact test was used to evaluate the relationship between DAD and death, and logistic regression was used for the DAD phase assessment of the histopathological lesions. *P*  value < 0.05 was accepted as statistically significant. Statistical evaluations were performed using SPSS software (version 11.5; SPSS, Inc., Chicago, IL, USA).

## 3. Results

We examined 66 individuals who were autopsied between 2006 and 2008 at the Institute of Forensic Medicine, Morgue Specialization Department and were diagnosed with DAD. Of these cases, 55 (83.3%) were men and 11 (16.6%) were women. The mean age of the cases was 53 years, the median was 54 years, and the range was 19–92 years.

Histopathological examination revealed that 50 (75.7%) cases were in the exudative phase, and 16 (24.2%) were in the proliferative phase. Table 1 summarises the distribution of the diffusion intensity of histopathological lesions according to phase.

Only the rate of alveolar exudate/oedema was significantly higher in the exudative phase cases (*P* = 0.003). The rates of alveolar histiocytic desquamation (*P* = 0.037), alveolar fibrosis (*P* = 0.017), chronic inflammation (*P* = 0.02), and alveolar fibrin (*P* = 0.001) were significantly higher in the proliferative cases than in the exudative cases. No significant difference between the distribution of exudative and proliferative phases was observed for other examined lesions. In contrast, in the logistic regression analysis, only the presence of alveolar fibrin as an independent variable was predictive of a diagnosis in favour of proliferative cases (*P* = 0.016). The severity of alveolar chronic inflammation and diffusion of fibrosis were mild, and no case showed strong positivity for either of these parameters (Table 1).

The distribution of the events causing death is shown in Table 2. The most frequently observed event types were physical trauma and fire (Table 2). Twenty-eight cases (41%) suffered from physical trauma, including seven cases of traffic and occupational accidents. A fat embolism that developed after physical trauma was detected in a proliferative-phase DAD case. The cause of death could not be identified in one case, which was referred to the supreme board. No significant difference was detected in terms of exudative and proliferative phases based on the classification of case types as physical trauma, burns, and other events.

## 4. Discussion

DAD is difficult to diagnose due to the severity of preoperative histopathological ARDS, but may be diagnosed at autopsy. DAD is seen more frequently in individuals >50 years of age [18]. Whereas hospital autopsy series have identified infection as the most common factor, 92% of deaths in DAD cases undergoing forensic autopsy are unnatural, which is consistent with the distribution in our cases, and trauma is reported as the most common cause [15, 19, 20].

DAD is an inflammatory change observed in the alveolocapillary complex [21]. Although lesions demonstrate a diffuse or patchy distribution in all areas of the lung, they may rarely be localised [22]. This disease has early exudative (acute), subacute proliferative (organising), and late fibrotic (chronic) phases [23–26]. HM development manifests 4-5 days after damage [27]. In forensic autopsies, Ferrer et al. [3] detected HM development in 12% of exudative-phase cases and 62% of proliferative-phase cases. In our study, the prevalence of HM occurrence was higher in the proliferative phase than in the exudative phase. In cases characterised as proliferative, which is a later stage than exudative, the high rate of HM presence confirms the aforementioned observations.

A second question that comes to mind is how is this lesion distribution affected by the fact that our cases were forensic autopsy cases? No difference in HM distribution has been reported in pulmonary and extrapulmonary DAD, [28] with “pulmonary DAD” referring to damages caused by intra-alveolar pathological effects, such as pneumonia, and “extrapulmonary DAD” referring to systemic inflammatory events, such as sepsis, and conditions causing damage induced by circulating cytokines. In other words, although DAD develops in different ways, pathological changes observed in these two groups are similar. Considering the event types in our cases, both pulmonary and extrapulmonary DAD cases were included in this series. Only post-fire DAD cases may represent pulmonary or extrapulmonary developmental risks. In our opinion, forensic autopsies may become an appropriate means for DAD studies.

As DAD is a common pathological change that may be caused by several factors, rather than a single disease-specific factor, DAD alone is not expected to allow the physician to determine cause of death. Antemortem medical and forensic information should be evaluated jointly [29, 30]. The range of events presented in our study is sufficient to demonstrate the variety of cases. In deaths caused by fire, the effects of hypoxia and various gases released during burning play an important role in the mechanism of death [31, 32]. Physical trauma may lead to respiratory distress in several ways [33]. Possible mechanisms include haemorrhage-induced hypoxia caused by trauma, effects of cytokines secreted in the posttraumatic period, effect on the respiratory centre if the trauma is in the head, effect on respiratory muscles or increase in pulmonary arterial pressure, and the occurrence of oedema [34–36]. Surgical operations constitute an important risk factor, since anaesthesia leads to respiratory ataxia and/or hypoxia, and the intervention itself has multiple negative effects on chest movement [37]. This condition is relevant, particularly for surgeries performed in the abdominal and thoracic areas [38]. ARDS development in drug intoxication cases may be associated with the direct toxic effect of the drug on alveoli, as well as with vascular changes created in the pulmonary system, acidosis formation, or induced cytokine release [39]. Alveolar damage may develop as a complication, particularly with chemotherapeutic agents, some antibiotics, and substances such as paraquat [40, 41]. All of these different mechanisms should be considered at autopsy and when identifying a possible cause of death. Particularly, in cases in which the cause of death was not a single main event, but a number of interrelated events, determining the reasons behind the changes in the lung may shed light on the cause of death and may provide a better assessment of the responsibilities of parties involved. In contrast, the mechanism of DAD development is not fully understood, [42] and DAD can be detected at autopsy in only half of individuals with a clinical picture of ARDS [18]. Therefore, even the absence of any DAD manifestation during autopsy would not rule out ARDS. Pathologists and forensic medicine specialists are required to have knowledge about possible mechanisms, as well as detailed information about the case, including clinical parameters.

Establishing a microscopic differential diagnosis is more important than associating the mechanism with the histological profiles of the cases. In this regard, the first group to be discussed in our study includes cases with alveolar organising fibrosis and alveolar fibrin. The HM was also present in varying proportions in all of these cases; therefore, our cases are not consistent with acute fibrosis and organising pneumonia, variants of DAD that lack the HM in alveolar gaps and show wide fibrin accumulation and alveolar organising fibrosis [43]. Underlying bacterial pneumonia can also be eliminated in the cases we presented, because of the small number of neutrophils in alveoli and septa [44]. In our opinion, the coexistence of alveolar septal fibrotic thickening and alveolar organising fibrosis observed in our cases is an identifier of the DAD proliferative phase. The low level of this activity in most cases also supports this point of view. For example, according to Kang et al. [45], interstitial and intra-alveolar fibroblastic activations are more common compared with other aetiologies in infection-related DAD. According to El-Zammar et al. [46], proliferative activities of fibroblasts in DAD cases are higher compared with usual interstitial pneumonia (UIP) and organising pneumonia. In particular, this recent study deserves further investigation, as it proposes a possible association with the mechanism.

Another condition included in the differential diagnosis is the acute exacerbation of UIP. These cases display the same histopathological reaction as DAD; however, patchy interstitial fibrosis and honeycomb-patterned changes in the background can also be identified during autopsy [42, 47]. In the present study, chronic inflammatory cells and alveolar septal fibrosis were not observed in either the exudative or the proliferative phase. None of the cases could be evaluated as an acute exacerbation of UIP because they did not meet the required pattern criteria, and these cases were inconsistent with DAD chronic fibrotic phases when criteria such as the mild severity of these two lesions, dominance of acute lesion features, and event characteristics were considered.

The differential DAD diagnosis also includes acute eosinophilic pneumonia (AEP) [48, 49]. Toxic inhalation, drug reactions, infections, parasites, mycoses, and smoking are among the aetiological factors of AEP. In this study, eosinophil infiltration of moderate severity was observed in one of the deaths caused by a burn injury. Additionally, the absence of eosinophilic microabscesses, diffuse HM, alveolar exudate, and type II pneumocyte hyperplasia led to the exclusion of AEP.

This study had some limitations. Alveolar haemorrhage was not considered in the histopathological examination because alveolar haemorrhage, passive congestion, and cellular degeneration in hypoxia that developed for various reasons are expected morphological findings at autopsy [50]. Information on the cases was obtained from autopsy reports. Information about the intervals between the cause of death, death, and intervention could not be obtained. The last bias is that the interobserver variability since different pathologists can have different opinions on the reported histopathological findings. 

Fatal traumatic events are predominant in DAD cases subject to forensic autopsy. The availability of detailed forensic and medical information on the development of the event in such cases would facilitate the establishment of a causal link and illuminate the sequence that caused death. In contrast, when it is considered that not all individuals with DAD die and that some survivors may develop a restrictive pathology in the future, the examination of forensic cases should consider probable late trauma complications in the lung in some situations. The idea that a trauma could have caused lung restriction during the late period, leading to disability and even shortened lifespan, remains to be explored.

The number of forensic autopsies, which gradually increases year by year, has reached an annual average of 4000 in Istanbul today [51]. Among these, a significant number of DAD and HM cases have been reported. For example, Pakis et al. [20] detected acute lung damage at various levels of severity in 61/77 postintensive care deaths in a 5-year retrospective review. This autopsy series included deaths related to several events such as burns, intensive care treatment, aspiration, and head trauma and constitute a very appropriate setting for detecting DAD.

In conclusion, DAD exudative and proliferative lesions may be detected during forensic autopsies. However, the detection of all DAD morphological criteria with the same intensity is not always possible in each case. Particularly in trauma and fire cases, several factors such as sepsis and intoxication may play a role in the aetiology of DAD. Cases with traumatic causes are more common. In forensic autopsy cases, the cause of death must be determined to evaluate the event within the context of possible mechanisms and available histopathological findings during the final assessment. If antemortem information is obtained properly and the required consent is obtained from the relatives of the deceased, forensic autopsies may provide a favourable means for expanding our knowledge about acute lung damage, DAD, and interstitial lung disease.

## Figures and Tables

**Table 1:** Distribution of the diffusion intensity of histopathological lesions according to diffuse alveolar damage (DAD) phase.

Histopathological lesion	Stage	Diffusion				Total *n* (%)	*P *value
		None	Mild	Medium	Intense
Alveolar exudate/oedema (*n* = 61)	Exudative	1	15	32	2	49 (98)	0.003
	Proliferative	4	8	4	0	12 (75)	

Alveolar acute inflammation (*n* = 52)	Exudative	7	39	3	1	43 (86)	0.16
	Proliferative	7	9	0	0	9 (56)	

Alveolar fibrin (*n* = 49)	Exudative	17	25	8	0	33 (66)	0.01
	Proliferative	0	9	6	1	16 (100)	

Neutrophilic abscesses (*n* = 5)	Exudative	47	2	0	1	3 (6)	0.064
	Proliferative	14	0	2	0	2 (13)	

Histiocyte desquamation (*n* = 64)	Exudative	1	33	15	1	49 (98)	0.037
	Proliferative	1	4	10	1	15 (94)	

Alveolar epithelial cell desquamation (*n* = 64)	Exudative	2	35	12	1	48 (96)	0.061
	Proliferative	0	6	9	1	16 (100)	

Eosinophilia (*n* = 1)	Exudative	49	0	1	0	1 (2)	0.75
	Proliferative	16	0	0	0	0 (0)	

Hyaline membrane (*n* = 39)	Exudative	23	12	14	1	27 (54)	0.42
	Proliferative	4	6	6	0	12 (75)	

Type II pneumocyte hyperplasia (*n* = 26)	Exudative	32	14	4	0	18 (36)	0.41
	Proliferative	8	5	3	0	8 (50)	

Alveolar chronic inflammation (*n* = 42)	Exudative	22	28	0	0	28 (56)	0.20
	Proliferative	2	14	0	0	14 (88)	

Alveolar septal fibrosis (*n* = 8)	Exudative	47	3	0	0	3 (6)	0.017
	Proliferative	11	5	0	0	5 (11)	

Alveolar organising fibrosis (*n* = 16)	Exudative	50	0	0	0	0 (0)	0.00
	Proliferative	0	13	3	0	16 (100)	

**Table 2:** Causes of death and diffuse alveolar damage (DAD) phase distribution.

Causes of death	Total cases *n* (%)	Exudative phase	Proliferative phase
Fire	20 (30)	16	4
Physical trauma	21 (31)	15	6
Postoperative complication	3 (4)	2	1
Traffic accident	5 (7)	3	2
Occupational accident	2 (3)	2	0
Drug intoxication	2 (3)	1	1
Firearm	3 (4)	2	1
Mushroom poisoning	1 (1)	1	0
Corrosive substance intake	1 (1)	1	0
Sepsis	3 (4)	3	1
Sharp trauma	1 (1)	1	0
Alcohol intoxication	1 (1)	1	0
Morphine poisoning	1 (1)	1	0
Pesticide intake	1 (1)	1	0
Unidentified	1 (1)	1	0
